# Underlying causes of cryptogenic stroke and TIA in The Nordic Atrial Fibrillation and Stroke (NOR-FIB) Study – the importance of comprehensive clinical evaluation

**DOI:** 10.1186/s12883-023-03155-0

**Published:** 2023-03-21

**Authors:** B. Ratajczak-Tretel, A. Tancin Lambert, R. Al-Ani, K. Arntzen, G. K. Bakkejord, H. M.O. Bekkeseth, V. Bjerkeli, G. Eldøen, A. K. Gulsvik, B. Halvorsen, G. A. Høie, H. Ihle-Hansen, S. Ingebrigtsen, C. Kremer, S. B. Krogseth, C. Kruuse, M. Kurz, I. Nakstad, V. Novotny, H. Naess, R. Qazi, M. K. Rezaj, D. M. Rørholt, L. H. Steffensen, J. Sømark, H. Tobro, T. C. Truelsen, L. Wassvik, K. L. Ægidius, D. Atar, A. H. Aamodt

**Affiliations:** 1 Department of Neurology, Østfold Hospital Trust, Postboks 300, Grålum, 1714 Norway; 2 Institute of Clinical Medicine, University of Oslo, Oslo, Norway; 3 Department of Cardiology, Østfold Hospital Trust, Grålum, Norway; 4 Department for Neurology, Nordlandssykehuset, Bodø, Norway; 5 Lillehammer Hospital, Department of Neurology, Innlandet Hospital Trust, Lillehammer, Norway; 6 Research Institute of Internal Medicine, Oslo University Hospital, Oslo, Norway; 7 Department of Neurology, Molde Hospital, Molde, Norway; 8 Department of Internal Medicine, Diakonhjemmet Hospital, Oslo, Norway; 9 Stroke Unit, Oslo University Hospital, Ullevål, Oslo, Norway; 10 Department of Internal Medicine, Vestre Viken Hospital Trust, Baerum Hospital, Gjettum, Norway; 11 Department of Neurology, University Hospital of North Norway, Tromsø, Norway; 12 Department of Neurology, Skåne University Hospital, Malmö, Sweden; 13 Department of Clinical Sciences, Lund University, Lund, Sweden; 14 Department of Neurology, Vestfold Hospital, Tønsberg, Norway; 15 Department of Neurology, Herlev Gentofte Hospital, Herlev, Denmark; 16 Department of Neurology, Stavanger University Hospital, Stavanger, Norway; 17 Drammen Hospital, Department of Neurology, Vestre Viken Hospital Trust, Drammen, Norway; 18 Department of Neurology, Haukeland University Hospital, Bergen, Norway; 19 Department of Neurology, Telemark Hospital, Skien, Norway; 20 Department of Neurology, Rigshospitalet University Hospital, Copenhagen, Denmark; 21 Department of Neurology, Bispebjerg University Hospital, Copenhagen, Denmark; 22 Department of Cardiology, Oslo University Hospital, Ullevål, Oslo, Norway; 23 Department of Neurology, Oslo University Hospital, Rikshospitalet, Oslo, Norway; 24 Department of Neuromedicine and Movement science, The Norwegian University of Science and Technology, Trondheim, Norway

**Keywords:** Cryptogenic stroke, Stroke cause, Atrial fibrillation, Insertable cardiac monitor, Guidelines, Secondary prevention

## Abstract

**Background:**

Cryptogenic stroke is a heterogeneous condition, with a wide spectrum of possible underlying causes for which the optimal secondary prevention may differ substantially. Attempting a correct etiological diagnosis to reduce the stroke recurrence should be the fundamental goal of modern stroke management.

**Methods:**

Prospective observational international multicenter study of cryptogenic stroke and cryptogenic transient ischemic attack (TIA) patients clinically monitored for 12 months to assign the underlying etiology. For atrial fibrillation (AF) detection continuous cardiac rhythm monitoring with insertable cardiac monitor (Reveal LINQ, Medtronic) was performed. The 12-month follow-up data for 250 of 259 initially included NOR-FIB patients were available for analysis.

**Results:**

After 12 months follow-up probable stroke causes were revealed in 43% patients, while 57% still remained cryptogenic. AF and atrial flutter was most prevalent (29%). In 14% patients other possible causes were revealed (small vessel disease, large-artery atherosclerosis, hypercoagulable states, other cardioembolism). Patients remaining cryptogenic were younger (*p* < 0.001), had lower CHA_2_DS_2_-VASc score (*p* < 0.001) on admission, and lower NIHSS score (*p* = 0.031) and mRS (*p* = 0.016) at discharge. Smoking was more prevalent in patients that were still cryptogenic (*p* = 0.014), while dyslipidaemia was less prevalent (*p* = 0.044). Stroke recurrence rate was higher in the cryptogenic group compared to the group where the etiology was revealed, 7.7% vs. 2.8%, (*p* = 0.091).

**Conclusion:**

Cryptogenic stroke often indicates the inability to identify the cause in the acute phase and should be considered as a *working* diagnosis until efforts of diagnostic work up succeed in identifying a specific underlying etiology. Timeframe of 6-12-month follow-up may be considered as optimal.

**Trial registration:**

ClinicalTrials.gov Identifier NCT02937077, EudraCT 2018-002298-23.

## Background

Optimal secondary stroke prevention aiming to reduce stroke recurrence depends on the correct identification of the underlying etiology, and should be the fundamental goal of modern stroke management. Despite advances in the understanding of stroke pathophysiology and diagnostic techniques, cryptogenic stroke (CS) still accounts for 25 to 40% of ischemic strokes (IS) [1]. The category cryptogenic is heterogeneous, including cases with unknown etiology, two or more possible competing causes, or incomplete investigation [2–5]. It has been previously postulated that a large proportion of CS is of thromboembolic origin (embolic stroke of undetermined source, ESUS), with high suspicion of occult atrial fibrillation (AF) [6, 7]. Other possible causes include embolism due to patent foramen ovale (PFO) or cardiopathy, occult atherosclerosis from unstable plaques, and hypercoagulable conditions [8]. Empiric strategies for optimal secondary prevention in CS are unfortunately lacking and previous studies did not support routine administration of oral anticoagulation (OAC) in patients with ESUS [9, 10]. The best therapy to prevent stroke recurrence still depends on the correct identification of the underlying etiology [11, 12]. Considering recent years’ clinical trials results, developments in cardiology and neuroradiology, and the health economy perspective the focus should be placed on identifying high-risk conditions which may improve secondary prophylactic treatment.

The main purpose of the Nordic Atrial Fibrillation and Stroke (NOR-FIB) Study was to detect and quantify AF in patients with CS or cryptogenic TIA using an insertable cardiac monitor (ICM) and to identify biomarkers useful in clinical practice as predictors of incident AF [13, 14]. The results regarding arrhythmia detection and ICM usage have recently been published [15]. In this paper we present the spectrum of probable or possible underlying causes of CS and TIA revealed during a 12-month follow-up and discuss the importance of proper evaluation of the underlying etiology.

## Methods

### Study design and outcomes

**Fig. 1:**
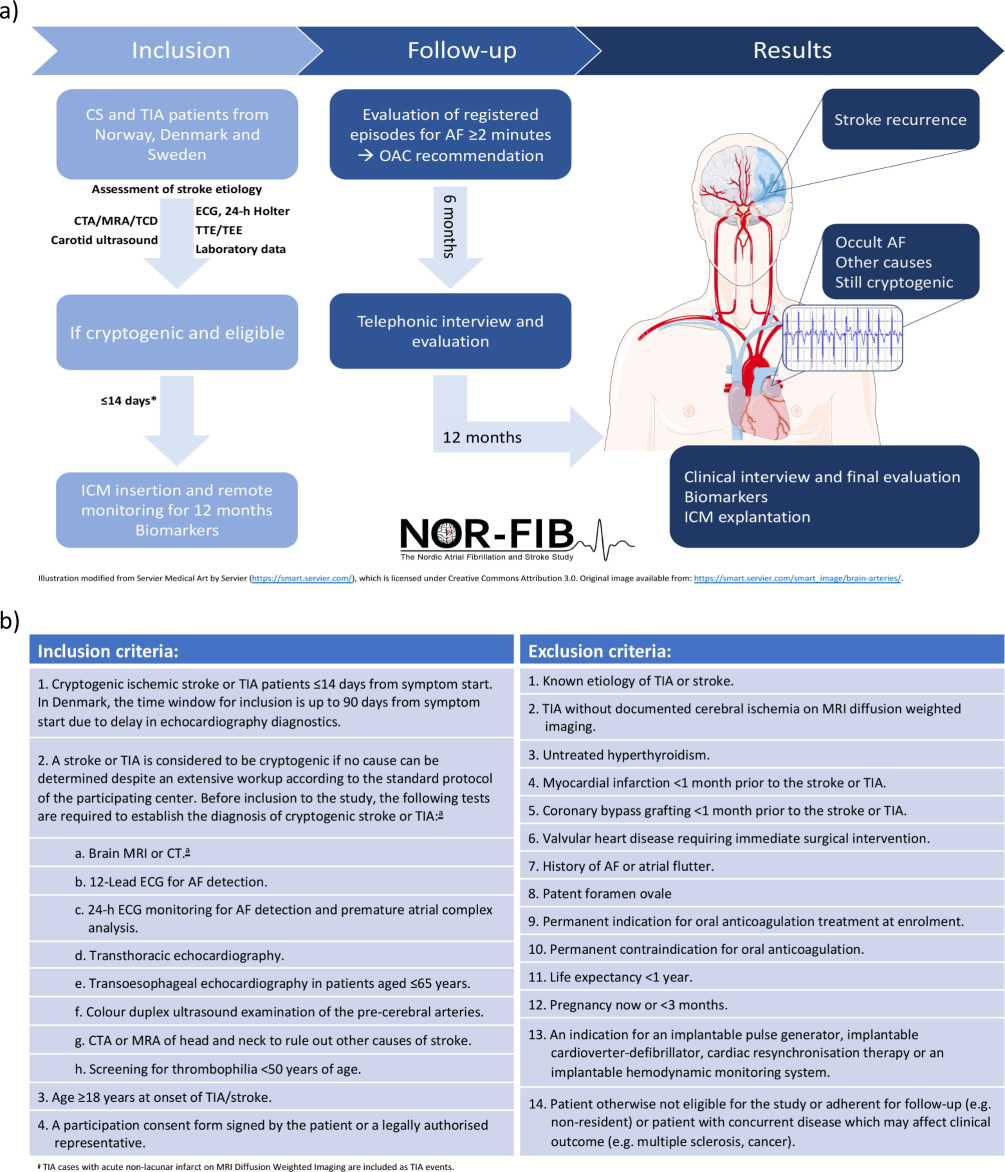
NOR-FIB study (a) study design diagram (b) inclusion and exclusion criteria

CS: cryptogenic stroke; TIA: transient ischaemic attack; CTA: computed tomography angiography; MRA: magnetic resonance angiography; TCD: transcranial doppler; ECG: electrocardiography; TTE: transthoracic echocardiography; TEE: transoesophageal echocardiography; ICM: insertable cardiac monitor; OAC: oral anticoagulation.

The NOR-FIB Study was an international, prospective, multicentre observational study of CS or cryptogenic TIA patients without previously documented history of AF monitored by ICM for 12 months for AF detection purpose. Patients in 18 participating centers from Norway, Denmark, and Sweden were included in the period from January 2017 to September 2020. The patients were examined by protocolled work-up before the diagnosis of CS or cryptogenic TIA was made (Fig. 1), as previously described [13]. CS was defined as a radiologically confirmed non-lacunar brain infarct in the absence of extracranial or intracranial atherosclerosis causing ≥ 50% luminal stenosis in arteries supplying the ischaemic area; major-risk cardiac source (including PFO) and any other specific cause of stroke. Similar criteria were previously used in the ESUS construct [6]. To avoid mimics, only clinical TIA cases with acute lesion on magnetic resonance imaging were included. All patients underwent 12-lead ECG and minimum 24-hour rhythm monitoring prior to enrolment to rule out AF or any other significant arrhythmia. One in three patients underwent AF screening ≥ 72 h monitoring. Transthoracic echocardiogram (TTE) was mandatory, while transesophageal (TEE) echocardiography was requested in patients ≤ 65 years. Completion of specified case report form (CRF) for echo data was optional. Measurements were done according to the current guidelines [16, 17]. Data for detailed patient description and blood samples for biomarkers analyses were collected at enrolment and at 12-month follow-up visit. Continuous cardiac rhythm monitoring was done by the Reveal LINQ® Medtronic device [18] in all patients. ICM monitoring was started early, median 9 (IQR 7–12) days after index event. AF episodes of ≥ 2 min duration, based on the detection algorithm of the device, resulted in change of secondary prevention from antiplatelet drugs to OAC. All patients were included in remote monitoring (ECG transmisions through distributed home monitors to CareLink network). ECG reports were weekly evaluated by a corelab, two neurologists and two cardiologists, to secure an early AF detection and start of anticoagulation.

The cryptogenic stroke diagnosis at enrolment and final diagnosis at 12-month follow-up was assessed by treating physician. The initial evaluation used in the NOR-FIB study reflected the clinical evaluation approach in the participating countries. The comprehensive evaluation strategy was used for AF detection only, while further evaluation for other underlying causes beside AF was in the discretion of patient’s physicians and oriented by clinical hints.

*Inclusion within 3 months from stroke onset was allowed for Danish centres.

### Statistical analysis

Data were censored at the time of death, study exit or completion of 12-month follow-up. IBM SPSS Statistics 26 software was used for the statistical evaluation. Categorical variables were presented as frequencies and percentages, and continuous variables as means and standard deviations (SDs) for normally distributed variables, and medians and interquartile range (IQR) for non-normally distributed variables. AF status of the patients with ICM monitoring time < 12 months (n = 5) was imputed according to the arythmia status at the time of the study dropout. Differences between groups were compared using Pearson Chi-Square or Fisher’s exact test for categorical variables according to data distribution, and Independent sample T-test or Mann-Whitney U-test for continuous variables. A *p* value < 0.05 was considered significant.

## Results

The 12-month follow-up data for 250 of 259 initially included NOR-FIB patients were available for analysis. Baseline patients’ characteristics are presented in Table 1. On admission stroke was diagnosed in 210 (84%) patients, whereas the remainder had clinical symptoms of TIA. The majority, 217 (86.8%) patients had initial symptoms indicating minor stroke (NIHSS score ≤ 5). Median pre-stroke vascular risk profile measured by CHA_2_DS_2_-VASc score was 2 (IQR 1–4). At discharge, previously undiagnosed hypertension was found in 37 (14.8%) patients, diabetes in 11 (4.4%) and dyslipidemia in 53 (21.2%) patients. Medical prophylactic treatment included acetylsalicylic acid in 187 (74.8%) patients, dipyridamole in 115 (46.0%), clopidogrel in 87 (34.8%), lipid lowering drugs in 228 (91.2%), and antidiabetic drugs in 22 (8.8%) patients.

**Table 1:** Differences between cryptogenic and non-cryptogenic patients on admission

	All included N = 250	Still cryptogenic N = 142	Etiology revealed N = 108	*p*-value
Age (years), mean (SD)	65.3 (12.6)	61.9 (13.0)	69.7 (10.7)	**<** 0.001*
Sex (%), female	41.2	40.8	41.7	0.896
Body mass index, mean (SD)	26.7 (4.4)	26.8 (4.6)	26.4 (4.3)	0.377
Acute stroke treatment (%)				
thrombolysis	26.0	27.5	24.1	0.545
thrombectomy	3.6	3.5	3.7	1
NIHSS, median (IQR)				
admission	1 (0–4)	1 (0–3)	2 (0–4)	0.161
discharge	1 (0–2)	0 (0–1)	1 (0–2)	0.031*
mRS score, median (IQR)				
admission	0 (0–0)	0 (0–0)	0 (0–0)	0.709
discharge	1 (0–1)	1 (0–1)	1 (0–2)	0.016*
CHA_2_DS_2_-VASc, median (IQR)	2 (1–4)	2 (1–3)	3 (1–4)	< 0.001*
Categories of risk (%),				
Low (0–1)	38.0	47.2	25.9	0.008*
High (≥ 2)	62.0	52.8	74.1	
**Comorbidity and risk factors (%)**				
Hypertension^1^	50.8	45.8	57.4	0.068
Diabetes mellitus^1^	8.4	7.7	9.3	0.669
Dyslipidaemia^1^	31.2	26.1	38.0	0.044*
Previous stroke/TIA^1^	23.6	21.8	25.9	0.450
Heart failure^1^	1.2	0.0	2.8	0.079
Myocardial infarction^1^	6.0	5.6	6.5	0.780
Vascular disease^1^	8.8	6.3	12.0	0.115
Current smoking^2^	21.2	26.8	13.9	0.014*
Cancer^3^	6.0	4.2	8.3	0.175
Valvular disease^4,5^	30.0	23.9	38.0	0.014*
Left atrial enlargement^4,6^	23.5	25.8	21.7	0.594
Left ventricle hypertrophy^4^	64.2	55.7	75.3	0.003*
**Medications (%)**:				
ASA	25.6	24.6	26.9	0.692
Dipyridamole	8.8	7.0	11.1	0.261
Clopidogrel	5.2	2.8	8.3	0.052
Diuretics	14.4	14.1	14.8	0.871
ACE inhibitors	9.2	4.9	14.8	0.007*
ARBs	23.2	22.5	24.1	0.775
Beta blockers	16.0	11.3	22.2	0.019*
CCBs	15.6	14.1	17.6	0.449
Antiarrhythmic drugs	1.2	0.0	2.8	0.079
Lipid lowering drugs	28.0	23.2	34.3	0.055
Oral antidiabetics	6.4	4.9	8.3	0.276
Insulin	2.4	2.1	2.8	1
Hormonal contraception	1.6	2.8	0.0	0.136
HRT	5.6	6.3	4.6	0.561

NIHSS: The National Institutes of Health Stroke Scale; CHA_2_DS_2_-VASc: Congestive heart failure, Hypertension, Age ≥ 75 years, Diabetes mellitus, prior Stroke or TIA or thromboembolism, Vascular disease, Age 65 to 74 years, Sex category; mRS: modified Rankin Score; ASA: acetylsalicylic acid; ACE: angiotensin-converting enzyme; ARB: angiotensin receptor blockers; CCBs: calcium channel blockers; HRT: hormonal replacement therapy

^1^self-reported or use of medication at stroke or TIA onset

^2^current smoking or if stopped < one year ago

^3^previous or current

^4^evaluated on echocardiography

^5^any type or grade

^6^moderate or severe

*p-value < 0.05

**Fig. 2:**
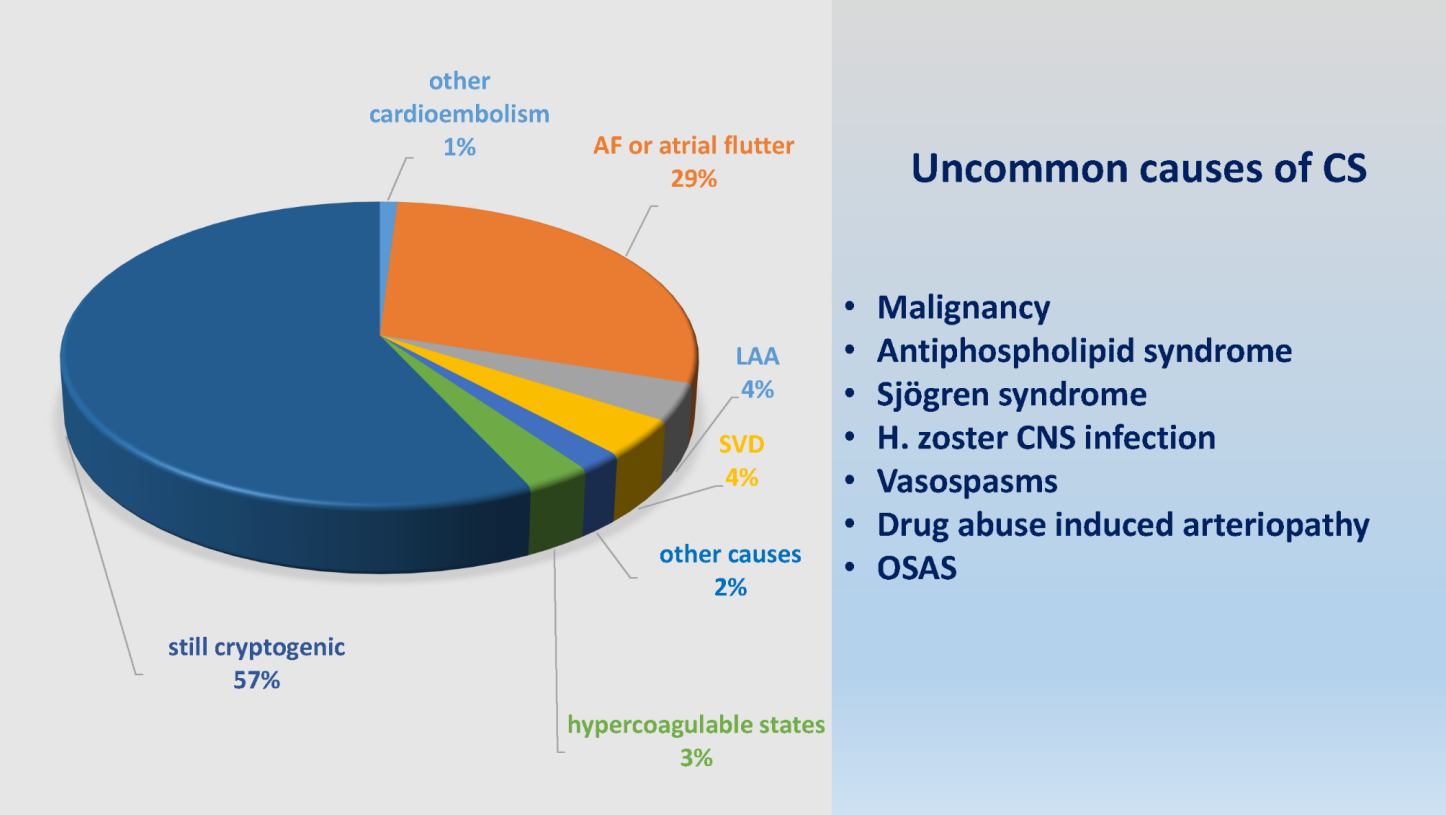
Heterogenity of CS and TIA etiologies in the NOR-FIB study

AF: atrial fibrillation; LAA: large-artery atherosclerosis; SVD: small vessel disease; H.zoster: Herpes zoster; CNS: central nervous system; OSAS: obstructive sleep apnea syndrome.

After the 12-month clinical visit and completing ICM monitoring, a broad spectre of probable or possible etiologies of stroke or TIA was revealed in 43% patients while 57% remained cryptogenic (Fig. 2). Paroxysmal AF or atrial flutter was detected in 74 patients (29%). Cardioembolism due to occult AF was considered the underlying cause in 72 of patients with detected AF (97.3%) and was the most frequent revealed etiology of CS in our study. In the remaining two patients the arrhythmia was deemed to be related to acute myocardial infarction and aortic valve replacement due to stenosis. Other source of cardioembolism was considered as a possible explanation in additional four patients; including three patients with atrial flutter episodes < 2 min and one with aortic valve stenosis requiring replacement.

Besides cardioembolism, possible stroke causes were revealed in another 13%. The most frequent etiologies were large-artery atherosclerosis (11 patients) and small vessel disease (10 patients). Hypercoagulable states due to antiphospholipid syndrome, elevated antiphospholipid antibodies and malignancy, were considered a probable or possible etiology in 7 patients. Other rare causes were seen in 5 patients.

Patients remaining cryptogenic after 12-month follow-up were younger (61.9 vs. 69.7 years; *p* < 0.001), had lower vascular risk assessed by CHA_2_DS_2_-VASc score (median 2 vs. 3; *p* < 0.001) on admission, and lower NIHSS score (median 0 vs. 1; *p* = 0.031) and mRS (*p* = 0.016) at discharge. Smoking was more prevalent in patients remaining cryptogenic (26.8 vs. 13.9%; *p* = 0.014), while dyslipidaemia (26.1 vs. 38%; *p* = 0.044) was less prevalent. Cryptogenic patients had also lower prevalences of valvular disease (*p* = 0.014) and hypertrophy of left ventricle (*p* = 0.003). However, AF patients were older (72.5 vs. 62.3 years; *p* < 0.001), had higher pre-stroke CHA_2_DS_2_-VASc risk score (median 3 vs. 2; *p* < 0.001), NIHSS on admission (median 2 vs. 1; *p* = 0.003) and discharge (median 1 vs. 0, *p* = 0.014) compared to patients not having AF. Valvular disease (*p* = 0.031), left ventricle hypertrophy (*p* = 0.030) and dyslipidaemia (*p* = 0.006) were more prevalent, while smoking (*p =* 0.011) less prevalent in AF patients. At 12-month control cryptogenic patients had persistent lower vascular risk profile (CHA_2_DS_2_-VASc score 4 vs. 5; *p* < 0.001) and cancer rate (4.2 vs. 12.0%; *p* = 0.021).

OAC was recommended to all patients with verified AF or atrial flutter and at 12-months` follow-up 97.3% were on this therapy. Secondary prevention was also optimized in patients where other underlying causes were found. Stroke recurrence was higher in the group remaining cryptogenic compared to the group with etiology revealed, eventhoug the difference was not significant, 7.7% vs. 2.8% (*p* = 0.091) probably due to short follow-up time. For the AF group stroke recurrence was 2.7% vs. 6.8% for non-AF patients (*p* = 0.363*)*, yet no stroke reoccurred after OAC initiation in the AF patients (Table 2).

**Table 2:** Differences between cryptogenic and non-cryptogenic patients at 12-month follow-up

	All included N = 250	Still cryptogenic N = 142	Etiology revealed N = 108	*p*-value
CHA_2_DS_2_-VASc, median (IQR)	4 (3–5)	4 (3–4)	5 (4–6)	< 0.001*
**Comorbidity (%)**:				
Hypertension^1^	58.0	53.5	63.9	0.1
Diabetes mellitus^1^	10.4	9.9	11.1	0.591
Heart failure^1^	3.2	0.7	6.5	0.01*
Myocardial infarction^1^	0.8	0.7	0.9	1
Vascular disease^1^	10.4	9.2	12.0	0.460
Current smoking	12.4	15.5	8.3	0.089
Cancer^2^	7.6	4.2	12.0	0.021*
Cerebral hemorrhage	1.2	2.1	0.0	0.261
**Medications (%)**:				
ASA	34.0	44.4	20.4	< 0.001*
Dipyridamole	25.2	36.6	10.2	< 0.001*
Clopidogrel	29.6	41.5	13.9	< 0.001*
OAC	38.0	12.7	71.3	< 0.001*
Diuretics ACE inhibitors	11.2 12.4	12.7 9.2	9.3 16.7	0.396 0.074
ARBs	30.8	32.4	28.7	0.442
CCBs	21.2	17.6	25.9	0.111
Antiarrhythmic drugs	14.0	9.9	19.4	0.015*
Lipid lowering drugs	84.4	84.5	84.3	0.957
Oral antidiabetics	9.2	8.5	10.2	0.810
Insulin	2.4	2.8	1.9	0.701
Hormonal contraception	0.0	NA	NA	NA
HRT	3.6	3.5	3.7	1
Recurrent stroke or TIA, (%)	5.6	7.7	2.8	0.091

CHA_2_DS_2_-VASc: Congestive heart failure, Hypertension, Age ≥ 75 years, Diabetes mellitus, prior Stroke or TIA or thromboembolism, Vascular disease, Age 65 to 74 years, Sex category; ASA: acetylsalicylic acid; ACE: angiotensin-converting enzyme; ARB: angiotensin receptor blockers; CCBs: calcium channel blockers; HRT: hormonal replacement therapy

^1^self-reported or use of medication at 12-month control

^2^previous or current

*p- value < 0.05

Interestingly, 12 patients in cryptogenic group had echocardiography findings of medium-risk sources of embolism (hypokinetic left ventricular segment, mitral valve prolapse, mitral annulus calcification and atrial septal aneurysm), neglected by treating physicians as possible CS etiology on both initial and final evaluation.

Table 2. Differences between cryptogenic and non-cryptogenic patients at 12-month.

follow-up.

## Discussion

In the NOR-FIB study probable or possible etiology was revealed in almost 1 of 2 patients previously classified as cryptogenic by extending the follow-up period to 12 months and implementing continuous long-term cardiac rhythm monitoring with the ICM for AF detection purpose. Our findings suggest that, when the etiology is not revealed in the acute phase the diagnoses cryptogenic stroke and cryptogenic TIA should be considered as *working diagnoses* until efforts of diagnostic work up succeed in identifying an underlying etiology. This is specially true due to lack of guidelines for standard evaluation maintained to conclude wether the stroke is of undetermined etiology. Timeframe of 6 or 12-month may be considered as optimal observational period for underlying AF as well as other diseases stroke may be the first manifestation of (i.e. malignancy or antiphospholipid syndrome).

As assumed, occult AF occurred in a substantial part of CS patients extensively monitored for this purpose. It is widely known that the duration of monitoring needed to detect paroxysmal arrhythmias seems to be inversely proportional to arrhythmia burden, so to properly rule out paroxysmal AF longer monitoring is needed. Current knowledge suggest that up to one in three CS patients may be diagnosed with AF using prolonged cardiac monitoring [19]. ICMs are the most effective tools revealing AF in 16–34% of the CS patients [20]. Nevertheless, as we are still awaiting randomized controlled trials confirming reduced risk of stroke recurrence after subclinical AF detection, ICMs have so far been rather rarely offered to eligible patients, mainly due to the limited economic resources in many countries. Our study has clearly demonstrated ICM as a feasible tool for stroke physicians to manage and highly effective for diagnosing underlying AF [15]. Identification of the underlying arrhythmia to prevent stroke recurrence by anticoagulants or left atrial appendage closure [21] is important especially for patients with minor stroke or TIA, as in our population, in whom a new AF-related stroke may be more severe or even fatal. The World Stroke Organization (WSO) Global Stroke Services Guidelines and Action Plan [22] and European Stroke Organisation (ESO) Stroke Action Plan for Europe 2018–2030 [23], focusing on feasibility of comprehensive approach in stroke care, emphasize the role of effective secondary prevention applicable to almost all IS and TIA patients. Furthermore, the latest *ESO guideline on screening for subclinical atrial fibrillation after stroke or transient ischaemic attack of undetermined origin* recommends early start and longer duration of cardiac rhythm monitoring of more than 48 h with ICM to increase the detection of subclinical AF [24]. This recommendation, long awaited among stroke physicians, is a step toward better IS evaluation and will hopefully contribute to reducing the proportion of events misclassified as cryptogenic if complied with.

Beside AF or atrial flutter, most NOR-FIB patients did not experience any significant arrhythmia or cardioembolism indicating OAC usage. The last is probably due to extensive echocardiography usage where all patients were screened for major-risk cardiac sources prior enrollment [13]. However, as discovered, 12 patients in group still remaining cryptogenic had initial echocardiography findings of medium-risk sources of embolism that were unrecognized as a probable stroke cause. Sufficient cardiac evaluation and its *correct* interpretation is an undeniable factor that helps to classify IS properly [2, 25]. Interestingly, 8% of CS patients were reclassified as large-artery atherosclerosis (LAA) or small vessel disease (SVD) strokes within 12 months follow-up. Those two main subtypes of IS were the second and third most frequent cause after cardioembolism in our study. The reassessment might be a consequence of excluding other causes during the follow-up time (particularly AF), or if misclassification in the acute phase. Increasing awareness on proper initial radiological evaluation is another target to improve stroke diagnostics. Atherosclerosis with < 50% vessel stenosis in precerebral arteries or plaques in the aortic arch and thoracic aorta is now being considered as potential cause of CS [26]. However non-significant vessel stenosis was already in updated TOAST (the Trial of ORG 10,172 in acute stroke treatment) classification SSS-TOAST, for near two decades ago, pointed out as a possible stroke mechanism [27]. Regarding SVD, in a small proportion of patients SVD may be due to rare genetic variants that should be considered in patient without obvious vascular risk profile [28, 29]. A wide range of other possible, uncommon causes were demonstrated in 5% patients. Identification of these, even if rare, is important to avoid inappropriate and expensive diagnostics (i.e. if vasospasm due to known migraine) and secure optimal treatment (i.e. antiphospholipid syndrome). Thrombosis and hemostasis abnormalities may play a key role in stroke in the young [30]. Thrombophilia tests may however be falsly abnormal in the acute phase and testing should be delayed for several weeks, for when a patient is off anticoagulation. Initially positive antiphospholipid antibody result need to be confirmed three months later. The recent COVID-19 pandemic shed light on the underlying mechanisms of infection- and vaccination-induced hypercoagulability leading to acute IS [31]. In three patients diagnosed with malignancy during the follow-up period, the index stroke was retrospectively considered cancer-associated (probably its first manifestation). Underlying occult malignancy can, directly or indirectly, increase stroke risk due to tumor associated hypercoagulability, embolism, as well as elevated risk of AF and atherosclerosis due to cancer treatment [32]. Cryptogenic stroke patients have a higher risk of cancer diagnosis in the following 6–12 months [33]. Patients with active cancer and ESUS have several identifiable characteristics: except smoking fewer traditional stroke risk factors, increased D-dimer and inflammatory markers, more severe or embolic-appearing infarcts in bilateral anterior and posterior circulations [34]. Stroke risk is also elevated in cancer-survivors so reflection on all relevant risk factors is required in comprehensive stroke assessment [35].

Summarizing, there was great heterogeneity among the potential causes of CS, including atherosclerotic plaque, valvulopathies, hypercoagulable states, and others. Our findings are however in line with previous reports and cohort descriptions [36, 37], emphasizing that very rare causes cannot explain the frequency of CS, which is rather due to known risk factors going undetected as pointed out by Mohr for over 30 years ago [38]. This may also be reflected by the higher prevalence of vascular risk factors and considerable stroke recurrence rate at 5.6% for the whole NOR-FIB population, which is higher than previously reported for CS patients [4, 39].

With this paper we want to increase awareness on proper diagnostics of IS and TIA. One treatment option does not fit all CS patients as it not cover the different stroke subtypes and mechanisms. The NOR-FIB study results underscore the need for strengthening of stroke evaluation to secure final diagnosis in patients initially classified as cryptogenic. The best diagnostic approach include wide clinical expertise, good quality of cardiac and vascular imaging, and extended evaluation time if needed. The timeframe of 6- or 12-month follow-up may be considered necessary as not all underlying conditions can be detected immediately.

### Limitations

One of the limitations is that the rate of underlying, but not revealed causes may have been even higher. Unfortunately, there is no way to steadfastly establish the etiology of IS fulfilling Hills criteria for causality, and diagnostic criteria for different stroke subtypes represent only the balance of probabilities with respect to the etiology. However, the goal is to identify most likely etiology but not neglecting the possibility of other potential causes. The assessment of underlying cause was up to the discretion of local investigators both at baseline and follow-up. Study protocol stated that only patients without revealed etiology after protocolled work-up could be included. One might speculate whether all relevant causes were initially properly excluded, as previously explained. I.e., in one patient echocardiography showed aortic valve stenosis, but its association to CS was not commented on the final evaluation. Aortic valve stenosis may lead to atrial and ventricular remodeling, predispose to AF, and be an independent risk factor of IS [40]. As the focus in this study was cardioembolism and arrhythmia detection, no additional advanced diagnostics were required for atherosclerosis evaluation in patients with < 50% lumen stenosis in the relevant artery. Focused CTA re-assesment might have possibly revealed more of underlying large-artery atherosclerosis [37].

Another limitation is the assessment of lacunar strokes in the present study. We did not analyse raw MRI data so small bias may had arisen (as some of the initially CS were during follow-up reclassified as SVD). Lacunar strokes, however, may also occur in patients with AF and small cardiac embolies.

Finally, the sample size of the study and follow-up time may not show the real difference for stroke risk recurrence in favour of OAC treated AF patients. This may also be true for the NOR-FIB patients remaining cryptogenic, not having any significant arrhythmia and probably at lower risk of cardioembolic stroke and stroke recurrence in general. However, all patients were followed to detect also other causes than AF so optimized secondary prevention may have lowered recurrence risk in both groups.

### Future perspectives

There is a need to optimize work-up to identify the etiology in a larger proportion of CS and cryptogenic TIA patients. Specific guidelines for CS evaluation and treatment are still lacking, except the recent ESO guideline on AF screening and the ESO PFO management guidelines in development. Algorithms for standard and advanced stroke and TIA evaluation to avoid overdiagnosing CS may be of benefit while waiting for guidelines [41, 42]. Data-driven machine-learning analyses identifying subgrups of CS patient strongly associated with arterial disease, atrial cardiopathy, PFO, left ventricular disease or cancer may also help optimize secondary prevention [43].

With better access to key investigational modalities in the acute phase, awareness on stroke mechanisms and a more extensive evaluation with an individualized approach in extended phase, the etiology can be revealed in a higher proportion of patients. Implementation of the newest ESO guidelines on AF detection will hopefully contribute to equity of access and equality of stroke care. Extended use of ICM and imaging diagnostics may not only contribute to etiology detection but also clarify patients with the lowest recurrence risk (an important, unmet need among stroke survivors). Next step would be to explore if a more extensive diagnostic work-up and extended follow-up time lead to fewer recurrent strokes in CS.

## Conclusion

Based on our findings, a significant proportion of IS and TIA caused by underlying conditions is still erroneously classified as cryptogenic, because standard evaluation done in the acute phase is often insufficient to reveal potentially underlying cause. Considering the term cryptogenic as a *working diagnosis* may contribute to a paradigm shift ensuring stroke patients optimal secondary prevention. Tailored treatment of underlying conditions can reduce the stroke recurrence significantly, so attempting a correct diagnosis should be the fundamental goal of stroke management in modern stroke units.

## Data Availability

The data that support the findings of this study are available, but restrictions apply to the availability of these data, which were used under license for the current study, and so are not publicly available. Data are however available from the authors upon reasonable request, for details please contact Anne Hege Aamodt (a.h.aamodt@medisin.uio.no).

## Declarations

### Concent for publication

Written informed consent was obtained from all participants.

## List of Abbreviation

AF: atrial fibrillation
ACE: angiotensin-converting enzyme
ARB: angiotensin receptor blockers
ASA: acetylsalicylic acid
CCBs: calcium channel blockers
CHA_2_DS_2_-VASc: Congestive heart failure, Hypertension, Age ≥ 75 years, Diabetes mellitus, prior Stroke or TIA or thromboembolism, Vascular disease, Age 65 to 74 years, Sex category
CNS: central nervous system
COVID-19: coronavirus disease of 2019
CRF: case report form
CS: cryptogenic stroke
CT: computed tomography
CTA: computed tomography angiography
ECG: electrocardiography
ESO: European Stroke Organisation
ESUS: embolic stroke of undetermined source
HRT: hormonal replacement therapy
H.zoster: Herpes zoster
IBM SPSS: International Business Machines Statistical Package for the Social Sciences
ICM: insertable cardiac monitor
IQR: interquartile range
IS: ischemic stroke
LAA: large-artery atherosclerosis
MR: magnetic resonance
MRA: magnetic resonance angiography
mRS: modified Rankin Score
NIHSS: The National Institutes of Health Stroke Scale
NOR-FIB: the Nordic Atrial Fibrillation and Stroke Study
OAC: oral anticoagulation
OSAS: obstructive sleep apnea syndrome
PFO: patent foramen ovale
SD: standard deviation
SVD: small vessel disease
TCD: transcranial doppler
TEE: transoesophageal echocardiography
TIA: transient ischemic attack
TOAST: the Trial of ORG 10,172 in acute stroke treatment
TTE: transthoracic echocardiography
WSO: World Stroke Organization
